# Echocardiographic analysis of the left ventricular function in young athletes: a focus on speckle tracking imaging

**DOI:** 10.11604/pamj.2016.25.171.9095

**Published:** 2016-11-16

**Authors:** Salma Charfeddine, Souad Mallek, Faten Triki, Rania Hammami, Dorra Abid, Leila Abid, Samir Kammoun

**Affiliations:** 1 Department of Cardiology, Hedi Chaker University Hospital, Sfax, Tunisia

**Keywords:** Athletes´ heart, speckle tracking echocardiography, strain

## Abstract

**Introduction:**

The objectives were to assess the left ventricular (LV) structure and function in regularly trained young athletes, using 2 D conventional echocardiographic (echo) methods and speckle tracking echocardiography (STE). An observational cross-sectional study.

**Methods:**

Thirty-three footballers and 20 healthy untrained subjects were included in the study. The systolic and diastolic LV functions were evaluated by 2D conventional echo parameters, Doppler method and STE.

**Results:**

All the found values were within the normal range. The LV End Diastolic Diameter (LVED 37.24±2.08 mm/m^2^) and the LV Mass index (LVMi 97.93±15.58 g/m^2^) were significantly higher in young athletes as compared with controls. There was no difference regarding the LV systolic function assessed by conventional echo parameters in the 2 study groups. Regarding the diastolic function, the transmitral inflow velocities ratio was significantly higher in athletes (E/A = 2.10±0.49 versus 1.64±0.26, p< 0.001) but there was no difference in the filling pressure in the 2 groups. The STE demonstrated a different pattern of LV deformation in the different groups. A significant lower LV global longitudinal strain (GLS -20.68±2.05 versus -22.99±2.32 %, p<0.001) and higher radial and circumferential strains have been found in the young athletes as compared with controls. A significant relationship between the GLS values and LVED (r= 0.299, p = 0.03) and LVMi was also reported in athletes.

**Conclusion:**

While conventional morphological and functional echocardiographic parameters failed to distinguish the adaptations in the athlete’s heart, deformation parameters showed a different pattern of LV mechanics in young footballers versus controls.

## Introduction

Athlete's heart is defined by all cardiac adaptations to intense physical exercise [1, 2]. Now, it is well known that participation in high-volume, high-intensity and regular sport activity results in significant morphological and functional modifications and remodelling of cardiac chambers. These central and peripheral cardiovascular adaptations facilitate the generation of a large and sustained cardiac output and enhance the extraction of oxygen from exercising muscle [2]. These morphological characteristics are currently assessed in athletes by two dimensional (2D) conventional echocardiographic parameters such as the left ventricular mass index (LVMi), the heart chambers diameters and wall thicknesses. In addition to the 2D standard echo parameters, assessment of myocardial function is currently possible by deformation parameters. The complex helical myofiber architecture of the LV is of a great clinical interest since deformation data in multiple directions provide valuable information in numerous pathological and physiological conditions [3]. The novel method of speckle tracking echocardiography (STE) allows us to quantify this mechanical aspect of the LV performance and also to investigate the different types of deformation separately [3, 4]. Investigations in elite athlete’s heart have recently provided relevant information on the LV deformation [5, 6]. However, little data concerning myocardial adaptation to regular training and LV deformation parameters in young elite athletes are available up until now [7, 8]. The aim of our study was to assess the LV structure and function in regularly trained young athletes (footballers), using 2 D standard echocardiographic methods and speckle tracking imaging.

## Methods

### Study population

A group of 33 young football players and 20 young untrained controls were enrolled. All the subjects were males. The athletes were selected among those who exercised = 6 hours/week on average for 3 years. Controls were selected among healthy individuals with sedentary lifestyle not involved in any sportive activities. None of the subjects had relevant findings as determined with physical examinations and medical history. All athletes were on a regular intensive exercise program. The study was approved by local Ethics Committee of Hedi Chaker university hospital of Sfax in Tunisia and oral informed consent was obtained before the participation to the protocol. For all the subjects (under 18 years old of age), oral parental consent was also obtained. After physical examination, different parameters were recorded for all participants: height, weight, heart rate and blood pressure. Body surface area (BSA) was calculated by the Mosteller formula [9]. The 2D transthoracic echocardiography (TTE), Doppler studies and STE were also performed using a GE Vivid E9 ultrasound machine.

### 2D standard echocardiography

For each subject included in the study, the echocardiographic measurements were performed at the left lateral position. The parasternal long-axis view was used for the M-Mode measurements of left atrial diameter (LAD), interventricularseptal, LV posterior wall thickness (IVST and LVPWT), LV end-diastolic and end-systolic dimensions (LVEDd and LVESd). In addition, the left atrial area and volume were determined. The LV fractional shortening (LVFS) was calculated as (LVEDd – LVESd)/LVEDd. The LV ejection fraction (LVEFs) was calculated from LV volumes by the modified biplane Simpson rule and expressed as a percentage.The evaluation of LV Mass Index g/m^2^ (LVMi) was obtained from Devereux procedure [10]. The pulsed Doppler transmitral flow velocity profile was obtained from the apical four-chamber view by the sample volume positioned below the mitral leaflets. Different parameters were evaluated: peak transmitral flow velocity in early diastole (E), peak transmitral flow velocity in late diastole (A), mitral A wave duration (dAm), E/A ratio, and the E deceleration time (DT).Analysis of pulmonary venous flow was realised at the right superior pulmonary vein. The duration of Ap wave (dAp) were measured. The difference between the pulmonary and mitral A waves duration (dAp-Am) was determined. Tissue Doppler imaging (TDI) was performed in the four-chamber view, with the mitral and tricuspid annular planes perpendicular to the ultrasound beam. Pulsed TD sample volume was placed at the septal and lateral aspects of the mitral annulus, and at the lateral aspect of the tricuspid annulus. Measurements were made of peak systolic (S’), peak early diastolic (E’), and late peak diastolic myocardial velocities (A’), and the E’/A’ratio at the lateral mitral annulus. The color M-mode Doppler flow propagation velocity (Vp) was obtained by combined color-flow Doppler and TM-mode interrogation of the mitral inflow during diastole.

### Speckle tracking echocardiography

The following six views were displayed: the apical four-chamber view, the apical three-chamber view, the apical two-chamber view and the three short-axis views, the apex of the LV, the midlevel of the LV, and the basal level of the LV. After tracing endocardial border at an endsystolicframe, the operator could validate the tracking quality and adjust the endocardial border or modify the width of the region of interest. Aortic valve opening and closure were selected on pulsed wave Doppler tracings recorded from the LV outflow tract. Frame rate ranged from 60 to 100 frame/s, and three cardiac cycles were stored in cineloop format for offline analysis. Global longitudinal LV strain (GLS) was defined as the average of negative longitudinal strains of 17 segments of the LV walls in the apical chamber views. Average LV radial and circumferential strain (GRS and GCS) was determined in the short-axis views.

### Statistical analysis

Statistical analysis was performed using Statistical Package for Social Sciences version 20.0 (SPSS, Chicago, IL, USA). Continuous variables with normal distribution were presented as the mean (standard deviation, SD). Qualitative variables were given as percent. Correlations between continuous variables were assessed using Pearson’s or Spearman’s correlation analysis. Two-way ANOVA with post-hoc analysis to compare the groups was used. A p-value under 0.05 was considered as statistically significant.

## Results

### Clinical characteristics

The average age of subjects was 13.19±1.2 years old in football players and 12.9±2.1 years old in control group. Subjects in the athlete group had a history of 4.75 ± 1.17 years of regular exercise and trained at least 11 months a year. The regime of the footballers consisted of 6 ± 0.89 training hours/week. The two groups were similar with respect to age, height, weight, body surface area (BSA) (m^2^), and systolic and diastolic blood pressures (SBP, DBP) (mmHg)(p> 0.05) (**Table 1**). The heart rate was significantly lower in the young athletes compared with controls (P < 0.001).

**Table 1:** Demographic data of the athletes and the control group

	Athletes (n=33)	Control group (n=20)	p
**Age (years old)**	13.19±1.2	12.9±2.1	0.13
**weight (Kg)**	38.27±6.5	38.0±20.2	0.95
**Height (cm)**	149.63±8.4	140.4±23	0.05
**BSA (m²)**	1.24±0.1	1.20±0.4	0.44
**SBP (mmHg)**	105.9±9.7	112.7±7.3	0.11
**DBP (mmHg)**	67.5±5.9	70.5±5.9	0.13
**Heart rate (pulse/mn)**	69.06±10.8	82.65±8.7	<0.001

Data are presented as mean ±SD, BSA: body surface area, SBP: systolic blood pressure, DBP: diastolic blood pressure

### Echocardiographic characteristics

#### 2D echocardiographic exam

LVEF were similar across the two groups and were within normal ranges (p = 0.13). LVED, LVES, and LA diameters were significantly larger in the athletes group compared with the controls (p<0.001, p = 0.03, and p = 0.02, respectively). IVST was significantly higher in the athletes compared with the control group (P = 0.02). Similarly, LVM index was significantly higher in the young football players than in the control group (P = 0.01) (**Table 2**).

**Table 2:** MM and 2D echocardiographic data of the athletes and the control group

	Athletes (n=33)	Controls (n=20)	P
LVED (mm/m²)	37.24±2.08	32.76±5.08	**<0.001**
LVES (mm/m²)	23.65±6.05	20.82±3.61	**0.03**
IVST (mm/m²)	7.37±1.49	6.51±1.22	**0.02**
LVPWT (mm/m²)	7.36±1.20	7.26±1.85	0.80
LAD (mm/m²)	25.06±2.93	22.69±4.65	**0.02**
EDV (ml/m^2^)	59.53±6.88	55.59±6.19	**0.04**
LVFS (%)	37.80±8.37	35.09±5.26	0.06
LVEF (%)	62.33±5.27	60.50±0.76	0.13
LA area (cm²)	11.93±3.26	9.04±4.58	**0.01**
LVM (g)	122.34±26.05	105.62±33.27	**0.04**
LVMi (g/m^2^)	97.93±15.58	84.74±23.65	**0.01**

Data are presented as mean ±SD, LVED: left ventricular end-diastolic dimension, LVES: left ventricular end-systolic dimension, IVST: interventricularseptal thickness, LVPWT: left ventricular posterior wall thickness, LVM: left ventricular mass, LVMi: left ventricular mass index, EDV: left ventricular end diastolic volume, LVFS: left ventricular fractional shortening, LVEF: left ventricular ejection fraction calculated by biplane Simpson method, LAD: left atrial diameter, LA area: left atrial area

#### Doppler parameters

Comparison of the standard transmitral Doppler parameters yielded similar E-wave and E-wave deceleration time for the 2 study groups. The A-wave was significantly lower in the athletes group (p<0.001) and the E/A ratio was significantly higher in athletes (p< 0.001). Comparison of TDI parameters measured from the lateral mitral annulus also demonstrated similar S’ and E’ rates for all 2 groups (p = 0.377and p = 0.177, respectively) (Table 3).

**Table 3:** Comparison of the Doppler parameters of the athletes and the control group

	Athletes (n=33)	Controls (n=20)	p
Transmitral Doppler parameters			
E-peak velocity (cm/s)	101.39±15.54	104.35±15.68	0.5
A-peak velocity (cm/s)	49.78±12.47	65.05±15.09	**<0.001**
E/A	2.10±0.49	1.64±0.26	**<0.001**
DT (ms)	152.61±40.24	168.00±37.04	0.17
TDI parameters in the lateral mitral annulus			
S’- peak velocity (cm/s)	12.39±1.59	11.60±1.93	0.11
E’-peak velocity (cm/s)	22.42±2.64	21.50±3.17	0.25
A’-peak velocity (cm/s)	5.75±0.79	7.80±2.50	**0.002**
E’/A’	3.96±0.73	2.89±0.59	**<0.001**
LV filling pressure			
E/E’	5.01±2.08	4.89±0.72	0.8
E/Vp	1.21±0.44	1.14±0.34	0.41
dAp-Am	-10.03 ±23.24	-14.25±19.17	0.34

Data are presented as mean ±SD

#### Speckle tracking analysis

**Figure 1:**
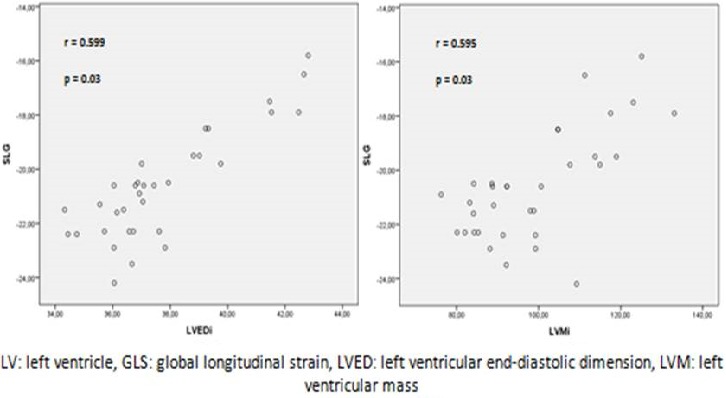
Relationship between LV GLS, LVED and LVM index in the athletes group

In this study, the LV longitudinal apical two, three and four-chamber strain and the LV GLS were significantly lower in the athletes as compared with controls (p = 0.002, 0.008, 0.007 and p< 0.001), whereas radial and circumferential strains were higher (Table 4). The segmental LV strain analysis yielded an increase in strain values from the basal to medium - apical segments, but there was a significant decrease of the strain values in the apical segments in the athletes as compared with controls (Table 5). There was a significant relationship between the LV GLS values, the LVED and the LVM index in the young footballers (r: 0.599, p = 0.03and r: 0.595, p = 0.03) (Figure 1).

**Table 4:** Comparison of the LV strain parameters of the athletes and the control group

	Athletes (n=33)	Controls (n=20)	P
S- 4C (%)	-19.10±2.46	-21.59±2.88	0.002
S- 3C (%)	-21.93±3.09	-24.42±3.37	0.008
S- 2C (%)	-20.94±2.56	-22.99±2.60	0.007
GLS (%)	-20.68±2.05	-22.99±2.32	<0.001
GCS (%)	-17.16±3.47	-16.40±3.22	0.43
GRS (%)	43.10±22.11	37.52±24.79	0.09

Data are presented as mean ±SD, S: longitudinal strain, 4C- 3C- 2C: apical four, three and two chamber views, GLS: global longitudinal strain, GCS: global circumferential strain, GRS: global radial strain

**Table 5:** Comparison of the segmental LV strain parameters of the athletes and the control group

Segmental LV strain		Athletes (n=33)	Controls (n=20)	p
Inferior	Basal	-20.81±3.72	-22.88±3.39	**0.04**
	Median	-23.39±2.92	-24.94±2.98	0.07
	Apical	-23.54±4.64	-25.98±4.00	**0.008**
Posterior	Basal	-19.90±5.00	-22.21±4.87	0.10
	Median	-22.12±5.85	-24.05±3.89	0.05
Anterior	Basal	-18.54±2.63	-18.77±4.36	0.81
	Median	-21.48±4.33	-22.63±4.66	0.36
	Apical	-21.18±4.93	-23.13±4.82	**0.001**
Antero-septal	Basal	-20.45±3.48	-18.99±10.01	0.44
	Median	-22.27±2.89	-23.98±5.05	0.12
	Apical	-21.30±3.46	-24.34±5.32	**<0.001**
Septal	Basal	-18.93±2.26	-20.25±2.08	**0.03**
	Median	-21.84±2.20	-22.42±2.44	0.38
	Apical	-21.45±2.82	-25.67±2.41	**<0.001**
Lateral	Basal	-18.48±6.17	-19.98±5.73	0.38
	Median	-19.90±4.66	-22.18±5.09	0.10
	Apical	-22.72±7.71	-25.00±4.86	**<0.001**

Data are presented as mean ±SD

## Discussion

Athletes develop different cardiac adaptations depending on the exercise program they use. The term “athlete’s heart” is used to describe the characteristic enlargement and hypertrophy of the myocardium in response to repeated exercise stimuli. To compensate the elevated cardiac output, the LV increases the internal diameters and secondary the LV wall thickness, cardiac pressure and heart rate [1]. Aerobic or endurance sports such as cycling, jogging are characterized by large muscle group dynamic exercise involvement, which subjected the LV to repetitive increase in cardiac preload, and presented ‘eccentric LV hypertrophy’. This hypertrophy was manifested as an increase in LV cavity dimensions and a proportional increase in LV wall thickness to normalize myocardial strain. In contrast, resistance- or strength-trained athletes are exposed to repetitive increases in peripheral vascular resistance and cardiac afterload. They showed ‘concentric LV hypertrophy’, manifested as increased LV wall thickness to normalize the increased wall tension associated with rise in pressure. In these resistance- trained athletes, there was little or no evident effect on cavity size. It was suggested that all these cardiac adaptations in athletes serve to normalise wall stress [11]. Most of the previous relevant studies in the literature included adults with very limited data concerning young athletes [7, 8, 12]. In the present study, we evaluated young football players. We assessed left ventricular dimensions, mass as well as systolic and diastolic functions with speckle tracking imaging study. In developing adult athlete’s heart, the LV septum wall thickness values of up to 13mm are usually considered as the physiological limit [13]. Values over 16 mm constitute the pathological range. Mean LV end-diastolic diameters have been reported as 53.6 ± 1.01 mm and mean LV mass as 214 ± 12.6 g [13]. These values are lower in younger athletes. In young elite athletes younger than 16 years of age, LV wall thickness was reported as 9.3 ± 1.3 mm and posterior wall as 9.6 ± 1.3 mm. LV end-diastolic diameter was described as 50.8 ± 3.7 mm, LV mass index as 113 g/m2 on average [14]. In this study, Sharma et al. reported that the LV wall thicknesses, LV end diastolic diameter and LVM were significantly higher in the young athletes than in the control group. In our study, the average LV septum wall thickness was 7.37±1.49 mm/m^2^, the LV ED diameter was 37.24±2.08 mm/m^2^ and LVMi was 97.93±15.58 g/m^2^.As reported in the literature, all these data were significantly higher in the young football players compared to the control group. In our study, the LV systolic function assessed by LVFS and LV EF was similar in the young footballers and the controls. Previous evaluations using this approach have demonstrated similar systolic functions between athletes and sedentary individuals [1]. The LV systolic function has most often been studied by use of conventional echocardiography and expressed as fractional shortening of the LV internal dimension or ejection fraction. Previous meta-analysis revealed that these indices of systolic function were usually not different between athletes at rest and matched control subjects [1, 13]. In some studies, however, fractional shortening or ejection fraction at rest was significantly higher or depressed in athletes, but these values were still within normal limits [13].

To highlight the young trained athletes’ heart, previous studies reported the effect of training on diastolic function in these children by using conventional Doppler examination of mitral flow patterns [15–19]. In the present study, peak E value was similar with no statistical difference between the young athletes and the non-trained boys. However, the late diastolic filling characteristics and the contribution of the atrial contraction (peak A velocity) was significantly lower in the trained children. The result of such modifications was the increase of E/A ratio in young athletes. Similar results were previously reported in some studies but not all [15, 16]. Other studies did not report enhancement in mitral inflow indexes in the trained state [7, 17–19]. Since transmitral inflow velocities are not only associated with the ventricular compliance but largely influenced by heart rate and preload conditions [20] , the training effect on myocardial function is difficult to interpret. TDI- derived velocity measurements have been suggested to be less affected by loading conditions than Doppler flow parameters. Few previous studies evaluated LV longitudinal velocities by TDI in children and controversial results were reported. Obert et al. [18] demonstrated that 2 months of endurance training failed to improve regional systolic and diastolic function evaluated by DTI in children. Ayabakan et al. [21] did not report differences in TDI measurements between pre-pubertal boy swimmers who had been training 8 hours per week for at least 3 years and age and sex-matched inactive controls. Rowland et al. [22] revealed no differences between young trained athletes and non-athletes in myocardial functional responses to progressive exercise evaluated by TDI measurements. Yet, a greater aerobic fitness in these athletes reflected volume expansion of the cardiovascular system without contribution of enhanced systolic or diastolic ventricular function. Such findings should be considered limited to the context of young athletes with limited duration of athletic training. However, Nottin et al. [16] established an improved diastolic function in 12–113-year old child cyclists who had been training 5–9 hours per week for at least 5 years than in non-trained age-matched controls. In the present study, there was no improvement in systolic LV function estimated by TDI velocity in the lateral mitral annulus. The LV diastolic function was improved in young trained footballers as a result of an increase in LV relaxation properties. But, the LV filling pressures were similar in trained individuals as compared with controls.

Although we identified similar LV systolic function conventionally (ejection fraction and TDI- peak systolic myocardial velocity), different supplementary methods may be needed to evaluate intrinsic myocardial functions in athletes with different LV geometry and diameters. So, more recent studies focused on the speckle tracking imaging for the assessment of the LV global and regional function. The STE or strain analysis is a relatively new and non-invasive imaging method based on tracking of characteristic speckle patterns created by interference of ultrasound beams in the myocardium, and has the advantage of measuring tissue velocities and deformation in an angle-independent fashion. The technique allows for simultaneous qualification in the long and short axes and quantification of regional wall motion abnormalities in all the myocardial segments [3]. Before the advent of STE, three dimensional deformation involving different in LV could be analysed only by magnetic resonance imaging (MRI), which is a costly and not always available in our daily practice. Recently, STE–derived measurements have been validated against sonomicrometry and tagged MRI, showing high feasibility and reproducibility [23]. There is little previous data concerning STE in young athletes’ heart. In the present study, although the LV systolic function was similar between the young footballers and the untrained healthy controls, we found that radial and transverse strains were higher in athletes. Whereas, despite being within the normal range, the LV global longitudinal strain was lower in our young athletes as compared with the control group. Furthermore, the regional LV strain analysis yielded an increase in the strain values from the basal to medium - apical segments, but there was a significant decrease of these values in the apical segments in the athletes as compared with controls. Conversely to our findings, Simsek et al. [7], who included in their study 22marathon runners, 24 wrestlers and 20 sedentary individuals, found that young athletes had higher values of global longitudinal strain than non-trained controls. De Luca et al. [8] analysed 50 young athletes (16 cyclists, 17 soccer players, 17 basket players) and 10 young controls. They conclude that the epicardial circumferential strain, the epicardial longitudinal strain, epicardial apex rotation and the LV twist were significantly higher only in cyclists. In the otherwise, Stefani et al. [24] reported that there was no significant difference in LV STE measurements between young athletes and control group. There are more studies using STE for evaluating adult athletes’ heart. Richand et al. [25] analysed 29 professional soccer players, 26 patients with hypertrophic cardiomyopathy (HCM) and 17 sedentary controls. They found that radial and transverse strains were significantly higher in athletes, whereas longitudinal strain was lower. However, compared to patients with HCM, professional athletes had higher values for transverse, radial and circumferential strains. Thus, the authors concluded that the lower value of global LV longitudinal strain observed in athletes (as compared to controls) could be a marker of a specific myocardial adaptation to the exercise-induced increase in volume overload, according to La Place’s law [25]. Recently, Caselli et al. [26] confirmed these findings in Olympic athletes, demonstrating that, despite being within the normal range, LV longitudinal strain is mildly lower compared with untrained controls. Conversely, further studies showed minimal or no differences in global LV longitudinal, radial and circumferential strains, as compared to healthy controls [27, 28]. Stefani et al. [29] in a study including 50 subjects (25 elite athletes and 25 sedentary controls), found that a brief isometric effort in athletes with physiological myocardial hypertrophy, produced significant enhancement of the strain in medium-apical LV segments as compared with controls. They concluded that these findings suggest the presence of a higher regional function reserve in the apical LV segments. In an athlete’s heart, it is crucial to determine the hypertrophy whether it is physiologic or pathologic. Indeed, LV hypertrophy in hypertension and HCM is related to sudden cardiac death and mortality, however, hypertrophy in athlete’s heart is accepted as a physiologic response. Thus, the STE can be considered as a helpful tool to differentiate diagnosis of physiological versus pathological LV hypertrophy. Many studies have been designed to assess differences in LV deformation in athletes and in patients with essential hypertension [28, 30] and HCM [25, 27]. All these studies conclude that the reduction in longitudinal strain is an early sign of LV dysfunction. Thereby, a significant reduction in LV GLS is an uncommon feature in athletes’ heart and shouldn’t be considered a physiological adaptation to training and an athlete presenting significant reduced LV strain should be carefully evaluated, particularly in the presence of an important hypertrophy [5]. Finally, data about myocardial deformation in young athletes remain limited up to now. Further larger studies using speckle tracking imaging are needed to highlight cardiac adaptation in young athletes.

### Study limitations

A limitation for our study was the relatively low case number of each group. The whole study population was consisted of male subjects. However, a previous study has demonstrated no gender-associated differences in strain imaging [30]. We used 2D speckle tracking analysis, whereas, it was recently reported the added value of 3D strain.

## Conclusion

This study evaluated LV systolic and diastolic functions in young athletes using conventional echocardiographic parameters and speckle tracking imaging method. Conventional echocardiography can detect some differences in trained young athletes’ heart but it fails to reveal differences in intrinsic myocardial functions. Speckle tracking echocardiography showed a different pattern of LV deformation in athletes versus untrained healthy subjects. Longitudinalstrain was lower in young athletes as compared with controls, whileradial and circumferential strains werehigher in athletes. Speckle tracking analysis canbe useful for identifying previously undetected differencesregarding the young athlete’s heart.

### What is known about this topic

- The athlete's heart is defined by all cardiac adaptations to intense physical exercise. The development of two-dimensional echocardiography led to important further advances to understand the cardiac modifications. Recently, novel echocardiographic techniques such as speckle tracking echocardiography provided further insights into the morphological and functional myocardial properties.
- The speckle tracking echocardiography is a relatively new non-invasive imaging technique that allows an objective assessment of the regional and global myocardial function.

### What this study adds

- Most of the previous relevant studies in the literature included adults with very limited data concerning young athletes. The present study highlights the importance of the echocardiographic study to detect progressive adaptations in the young athletes’ heart to regular sport activity and training.
- The conventional echocardiography can detect some differences in young athletes’ heart but it is incapable to reveal differences in intrinsic myocardial functions. Thereby, the evaluation of speckle tracking echocardiography can be useful for identifying previously undetected differences regarding athlete’s heart. The present study showed a lower left ventricular longitudinal strain in young athletes as compared with controls, while radial and circumferential strains were higher in athletes.
- Considering our results and the large heterogeneity of echocardiographic pattern generally found in adolescent subjects, several possible clinical applications of deformation parameters approach can be advised.
